# A Novel Neural Network-Based Technique for Smart Gas Sensors Operating in a Dynamic Environment

**DOI:** 10.3390/s91108944

**Published:** 2009-11-11

**Authors:** Hakim Baha, Zohir Dibi

**Affiliations:** Laboratoire d'Electronique Avancée, Département d'Electronique, Université de Batna, 05 Avenue Chahid Boukhlouf 05000 Batna, Algeria; E-Mail: zohirdibi@yahoo.fr

**Keywords:** gas sensor, ANN, implementation, ABM, corrector

## Abstract

Thanks to their high sensitivity and low-cost, metal oxide gas sensors (MOX) are widely used in gas detection, although they present well-known problems (lack of selectivity and environmental effects…). We present in this paper a novel neural network- based technique to remedy these problems. The idea is to create intelligent models; the first one, called corrector, can automatically linearize a sensor's response characteristics and eliminate its dependency on the environmental parameters. The corrector's responses are processed with the second intelligent model which has the role of discriminating exactly the detected gas (nature and concentration). The gas sensors used are industrial resistive kind (TGS8xx, by Figaro Engineering). The MATLAB environment is used during the design phase and optimization. The sensor models, the corrector, and the selective model were implemented and tested in the PSPICE simulator. The sensor model accurately expresses the nonlinear character of the response and the dependence on temperature and relative humidity in addition to their gas nature dependency. The corrector linearizes and compensates the sensor's responses. The method discriminates qualitatively and quantitatively between seven gases. The advantage of the method is that it uses a small representative database so we can easily implement the model in an electrical simulator. This method can be extended to other sensors.

## 1. Introduction

ANNs (artificial neural networks) are used in instrumentation to model complex systems because of their multivariable capability and strong non-linearity. With ANNs the extrapolation errors both inside and outside the calibration range are lower [1]. ANNs are very efficient for dynamic matter problems, offering the advantages of simple implementation and less computation demands, compared to numerical models [2].

Metal oxide sensors (MOX) are one of the most popular technological choices for sensor arrays, due to their high sensitivity [3]. Their main disadvantage is a lack of selectivity. The working principle of these sensors is based on the variation of their conductivity in the presence of oxidizing and reducing gases. The magnitude of the response depends on the nature and concentration of the gas, and on the type of metal oxide [4]. Researchers have examined some aspects of the non linearity of the MOX response to certain gases [5-7], like its dependence on the temperature [8,9], but the dependence on relative humidity and gas nature was not studied and no model of the MOX sensor has been implemented in a simulator.

The subject of linearization and compensation has been considered in different forms and stages; some cases studied include the use of analog to digital converters [10,11], while other work has focused on improving the nonlinear response of specific sensors, like the thermistor [12] and the Hall effect current sensors [13]. Numerical methods have been developed using modern technologies capable of computing linearization algorithms [14,15]. ROM memories are also used to save data tables and to solve the linearization problem [16]. They provide a limited solution to the complex problem under the assumptions that the range of variation of environmental parameters is small and that the influence of the environmental parameters on the sensor characteristics is linear.

To enhance the selectivity of metal oxide gas sensors various strategies have been used by different research groups, including the improvement of the sensitive material [17,18], the use of sensor arrays together with pattern recognition techniques [19,20], and measurements performed in dynamic operation mode [21,22]. In a dynamic environment these strategies don't take the influence of the environmental parameters into consideration or they provide a limited solution to the problem under the assumption that the range of variation is small and that influence on the sensor characteristics is linear.

**Figure 1.**
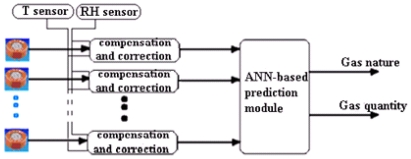
Multisensory system used.

For those purposes, using artificial neural networks we have designed a model and a corrector for each one of three industrial MOXs (TGS822, TGS821 and TGS813, by Figaro Engineering) and a selective module with the aim of improving the selectivity and eliminating the environmental effects. The diagram of the method used is presented in Figure 1. The MATLAB interface was used during the design phase and optimization; the results (optimal architecture, bias and weights of the network) are used for the implementation of the model, the correctors and the selective module as components in the PSPICE simulator library.

## 2. Sensor Characteristics

**Figure 2.**
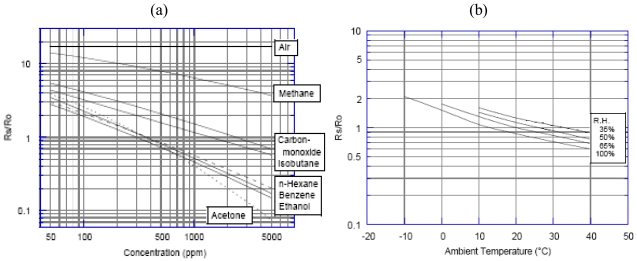
(a) TGS822 sensor's sensitivity feature [23]. (b) TGS822 dependence on temperature and relative humidity [23].

We illustrate the design of the TGS822 gas sensor model and the same procedure was followed for the TGS821 and the TGS813 units. According to experimental results [23], the TGS 822 sensor used to detect the gas concentration has a nonlinear sensitivity (Figure 2a; representation is in logarithmic scale) and is dependent on the temperature and humidity of the environment (Figure 2b) where it is placed. R_0_ is the sensor's resistance in 300 ppm of ethanol, while R_S_ the sensor's resistance to different concentrations of various gases.

### 2.1. The sensor's use

**Figure 3.**
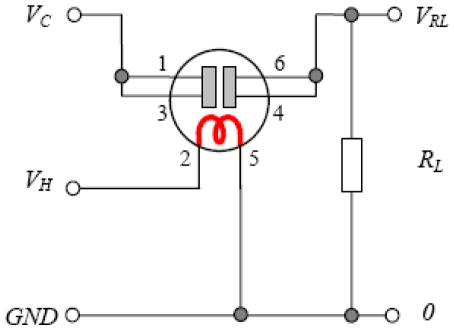
Measurement circuit with TGS sensor.

In Figure 3 a basic gas concentration measurement circuit with the TGS822 sensor is presented. The variation of the TGS sensor's resistance is indirectly measured by the voltage drop appearing on the reading resistance R_L_. If a gas like methane, butane, propane etc. comes into contact with the sensor's surface, it's resistance reduces in correlation with the gas concentration present. To work with the features of the sensor provided, in this case, by Figaro, we have to process this output voltage (V_RL_) and achieve the sensor's resistance R_S_ with Equation 1:

(1) $${\text{R}}_{\text{S}}=\left(\frac{{\text{V}}_{\text{C}}}{{\text{V}}_{\text{RL}}}-1\right)R_{L}$$

The configuration of the sensor's connected circuits has to ensure the following conditions:

- V_C_ can be 5, 6, 12 or 24 V.
- V_H_ heating voltage has to be 5 V ± 0.2 V.
- The power supply on the sensor maximum 15 mW.

If we determine the sensor's sensitivity feature under standard testing conditions, this characteristic has to coincide with the sensitivity feature, provided by the manufacturer, because this feature is determined with a relative representation of the sensor's resistance. Standard testing conditions, indicated by Figaro are:

- Atmospheric conditions: temperature 20 °C ± 2 °C and relative humidity 65% ± 5%
- V_C_: 10 ± 0.1 V, V_H_: 5 ± 0.05 V, R_L_: 10 K ± 1%
- Time for the sensor's supply maintenance: seven days or more
- Testing gas: ethanol

### 2.2. Sensor parameters

- Heating resistance: 38.0 Ω ± 3 Ω
- Sensor's resistance: 1∼10 KΩ at 300 ppm ethanol
- Resistance ratio:

(2) $$\frac{R_{S}\;\text{in}\;\text{Ethanol}\;300\;\text{ppm}}{R_{S}\;\text{in}\;\text{Ethanol}\;50\;\text{ppm}}=0.4\pm 0.10$$

Once the value R_0_ is measured, the sensor's resistance at different concentrations of various gases and different temperature and relative humidity will be determined as follows: with relation (1) we calculate R_S_, then from Figure 2b, for temperature and relative humidity, we read the value R_S_/R_0_ under the given conditions and we obtain a value *x*. We multiply the value *x* by R_0_ and we obtain a value *y*. We divide R_S_ by *y* and we get R_S_/R_0_, uninfluenced by temperature and relative humidity, with whose help, from Figure 2a, we will determine the appropriate concentration.

## 3. Neural Network Model

Using the MATLAB interface and based on experimental results from [23], according to the previous paragraph a database is created and arranged as (S, T, RH, C) input and (R_S_) as output where:

S: Selecting the gas
T: Absolute temperature
RH: Relative humidity
C: Gas concentration
R_S_: Sensor resistance
We suppose that Ro = 10 kΩ

**Figure 4.**
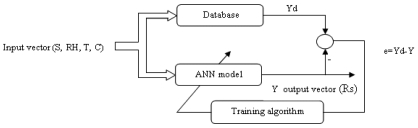
Modeling of the TGS822.

Most of this database is used mainly in the training phase by using the LP algorithm (back propagation of error), the remaining data are used to test and validate the model. The diagram in Figure 4 illustrates the direct modeling of the sensor, where:

Yd: Desired output
Y: Network output
e: Modeling error

To optimize the model architecture an iteration algorithm is used which consists in evaluation of the total error as a function of layer number and the number of neurons per layer and after many tests of different ANN models. The architecture optimized and that produces the smallest error is summarized in Table 1.

### 3.1. Model test

**Figure 5.**
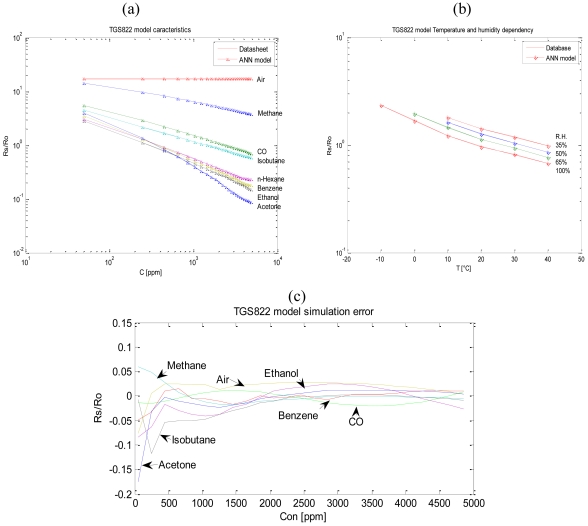
(a) Model and database TGS822 sensor's sensitivity feature. (b) Model and database TGS822 dependence on temperature and relative humidity. (c) Simulation error vs. concentration.

We designed a neural network-based model by taking into account the dependence on temperature and relative humidity at the measurement point, in addition to the dependence on gas nature when the sensor is placed in a dynamic environment. To illustrate this effect we change concentration, and then we note the variation of the sensor's resistance. Figure 5a shows the difference between the database and the ANN model for the sensor's sensitivity feature. Figure 5c shows the simulation error. The difference between the database and the ANN model for the dependence on temperature and relative humidity is shown in Figure 5b.

### 3.2. Implementation of TGS822 model

By using the ABM (Analog Behavioral Modeling) PSPICE Library components and the results (optimal architecture, bias and weights of the network) the sensor model designed previously is implemented as a component in the PSPICE simulator library.

**Figure 6.**
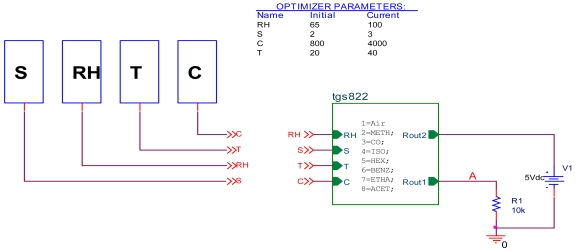
Simulation circuit.

In order to test the model introduced on PSPICE simulator, it has been implemented in the electrical circuit shown in Figure 6.

### 3.3. Simulation results

**Figure 7.**
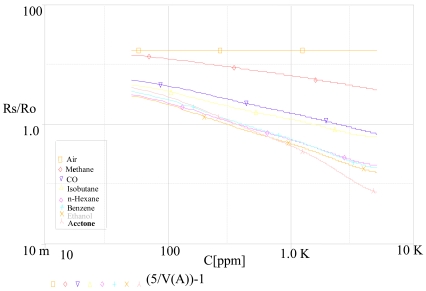
Variation of the résistance ratio (Rs/Ro) vs. gas concentration C at 20 °C and 65% RH.

Temperature is fixed at 20 °C, relative humidity at 65%, concentration is varied from 300 to 5,000 ppm. A PARAMETRIC DC SWEEP analysis gives the variation of the résistance against the concentration C with different gas. Results are represented in Figure 7 where:

- $Rs=(\frac{\text{V}1}{V(\text{A})}-1)\text{R}1$
- R1 = Ro = 10 kΩ

**Figure 8.**
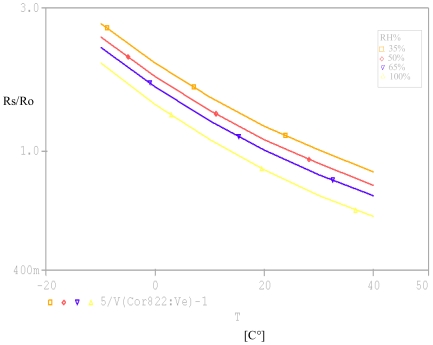
Variation of the résistance ratio (Rs/Ro) vs. temperature T at 300 ppm of ethanol.

By analogy to the first test, we carried out a second test. With a PARAMETRIC DC SWEEP analysis, we fixed the gas as ethanol, the concentration to 300 ppm and the relative humidity RH as a parameter then we varied the temperature from −10 to 40 °C. Figure 8 illustrates the obtained results.

## 4. Corrector

Using the circuit in Figure 3 and by analogy to the model we design the corrector. The database is arranged as (T, RH, V_RL_, V_S_) where:

T: Absolute temperature
RH: Relative humidity
V_RL_: Sensor's output voltage
Vs: Corrector's output voltage

**Figure 9.**
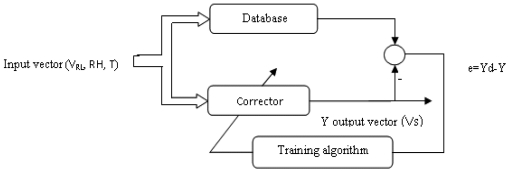
Modeling of the TGS822 corrector.

The generation of the training base and test base is similar to that of the model's one. However, in the corrector, the temperature T, relative humidity RH and the sensor's output voltage V_RL_ are taken as inputs, and the corrector's output voltage Vs is taken as output. The diagram of Figure 9 illustrates the methodology used in the corrector design.

The corrector was trained in a similar manner as in the case of the model. After many tests, the optimized architecture which produces the smallest error is summarized in Table 2.

### 4.1. Corrector test

**Figure 10.**
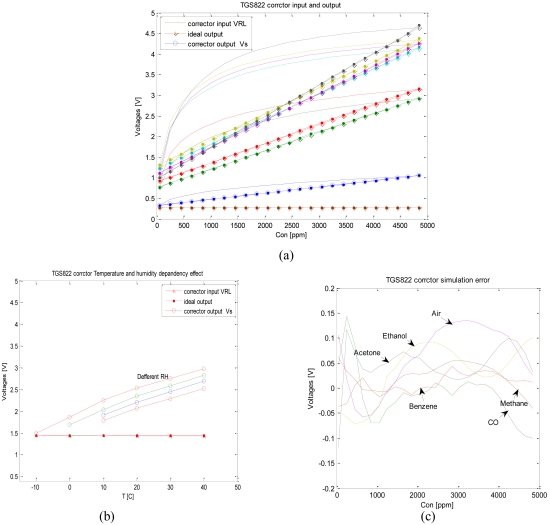
(a) TGS822 feature effect (b) TGS822 corrector temperature and relative humidity dependency effect (c) Corrector sensitivity simulation error.

We designed an ANN-based corrector for the TGS822. To illustrate the effect of this corrector we change concentration, and then we note the variation of the corrector's output. Figure 10a shows that the corrector correctly linearizes the sensor's sensitivity feature (representation is in linear scale). The corrector's effect in environmental parameters dependency is shown in Figure 10b.

### 4.2. Implementation of the TGS822 corrector

**Figure 11.**
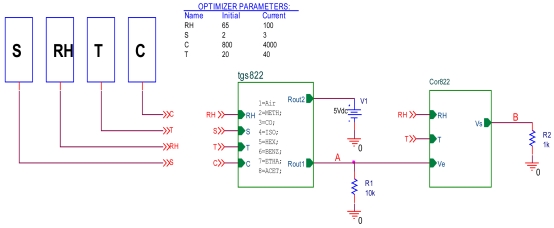
Electrical circuit of the corrector test.

In order to test the effect of the corrector on electrical environment, the latter is implemented on the PSPICE simulator. The corrector and the TGS822 model have been implemented in the circuit shown in Figure 11.

### 4.3. Simulation results

**Figure 12.**
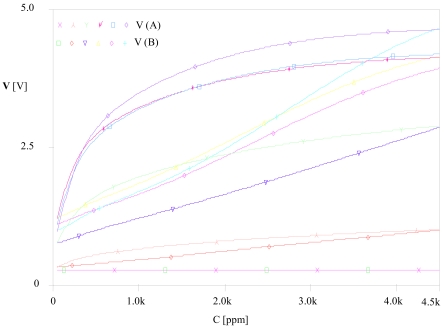
Variation of the corrector's input and output vs. concentration C at 20 °C and 65% RH.

Temperature is fixed at 20 °C, relative humidity at 65% and concentration is varied from 300 to 5,000 ppm. A PARAMETRIC DC SWEEP analysis gives the results represented in Figure 12 with different gases.

**Figure 13.**
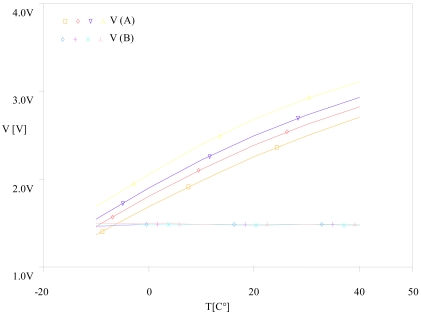
Variation of the corrector's input and output vs. temperature T at 300 ppm of ethanol.

The parametric simulation shows that the corrector linearizes the sensor's response whatever the gas. By analogy to the first test, we carried out a second test with a PARAMETRIC DC SWEEP analysis, we fixed the gas as ethanol, the concentration to 300 ppm and the relative humidity RH as a parameter then we varied the temperature from −10 to 40 °C. Figure 13 illustrates the obtained results.

The response of the parametric simulation shows that the corrector eliminates the environmental parameters' effect.

## 5. The Selective Module

By analogy to the model and the corrector we design the selective module. The database is arranged as (Ve1, Ve2, Ve3, Vsn, Vsc), where Ve1, Ve2, Ve3 are taken as input of the selective model and they are the output of the correctors, Vsn is taken as output and it defines the gas nature, Vsc is taken as output and it defines the gas concentration. Note that:

- Vcon is a voltage varing linearly from 0.4 to 5 V corresponding to a concentration variation from 400 to 5,000 ppm.
- Vsc is a voltage varing from 1 V to 10 V, the table 3 indicates their matched gases.

The selective module was trained in a similar manner as in the case of direct model and corrector. The optimized architecture characteristics are summarized in Table 4.

### 5.1. Implementation of the selective module

**Figure 14.**
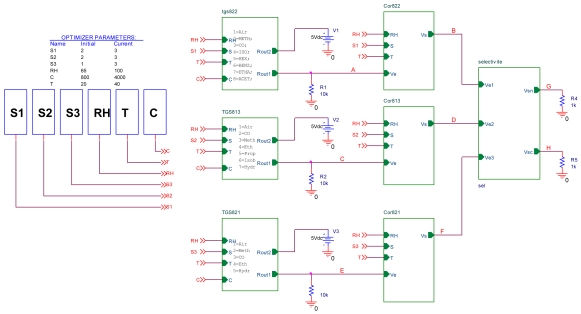
Electrical circuit of the method test.

The selective module was implemented in the same manner as the sensor model and the corrector. The schematic shown in Figure 14 represents the electrical circuit used for testing the technique used in electrical environment.

### 5.2. Simulation results

#### 5.2.1. Concentration effect

**Figure 15.**
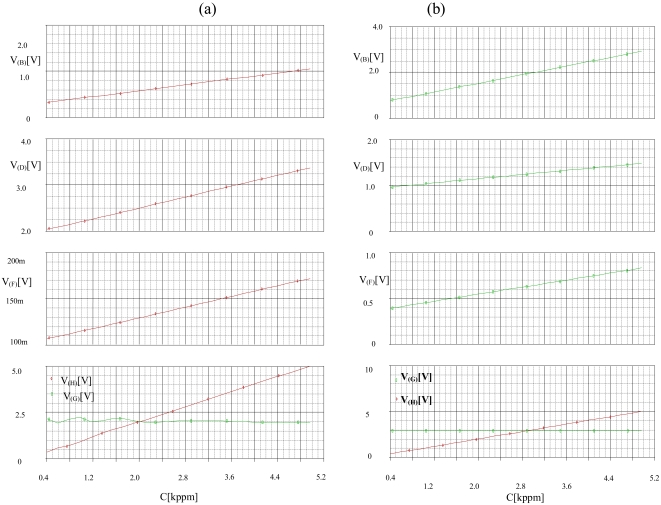
Variation of the inputs and the outputs the selective module vs. concentration C at 20 °C and 65%RH for the detection of (a) methane (b) CO.

**Figure 16.**
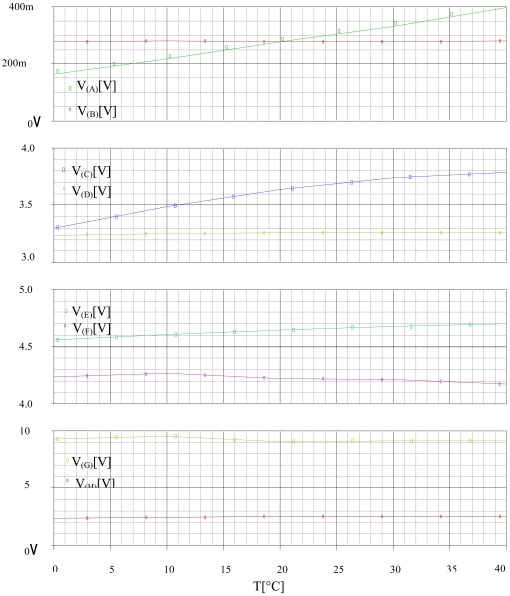
Variation of the corrector and the selective module's inputs and outputs vs. the temperature T at 65% RH for 2,500 ppm of hydrogen.

To study the concentration effect we fixed the temperature at 20 °C, relative humidity at 5% and concentration is varied from 300 to 5,000 ppm. A DC SWEEP analysis gives the results represented in Figure 15 for the choice of the methane and Figure 16 for the choice of the CO, where Ve1, Ve2 and Ve3 are the corrector output, Vsng is the selective module output for the gas nature and Vsc is the selective module output for the gas concentration.

Figure 15a shows that the method identifies the gas as methane (**V_(G)_** = 2V, Table 3), and the quantity of this gas (linear variation of **V_(H)_** against C). Figure 15b shows that the method discriminates the gas as CO (**V_(G)_** = 3V, Table 3), and the quantity of this gas (linear variation of **V_(H)_** against C).

#### 5.2.2. Temperature effect

The effect of the temperature is analyzed with a DC SWEEP analysis. 2,500 ppm of hydrogen is used for the test. We fixed the relative humidity to 65% then we varied the temperature from −10 to 40 °C. Figure 16 illustrates the results of this simulation.

Figure 16 shows that the method discriminates 2,500 ppm of hydrogen (**V_(H)_** = 2.5 V, **V_(G)_** = 9 V), and stay unchanged vs. the temperature.

#### 5.2.3. Relative humidity effect

**Figure 17.**
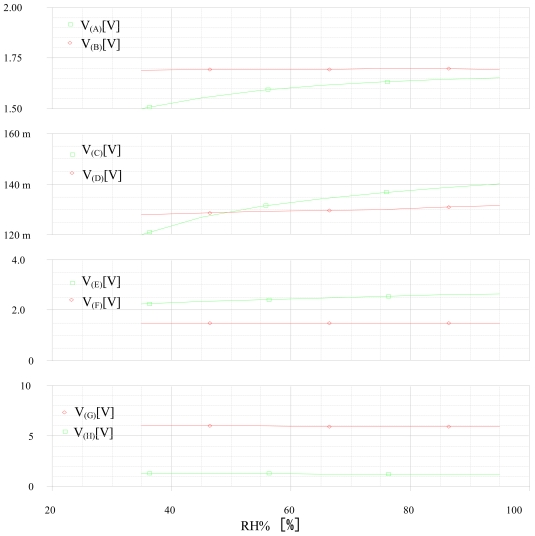
Variation of the inputs and the outputs of the correctors and the selective module vs. the relative humidity RH at 20 °C for the detection of 1,300 ppm of benzene.

We introduce the humidity effect with a DC SWEEP analysis. Figure 17 illustrates the relative humidity influence on the corrector and the selective module outputs.

Figure 17 shows that the method discriminates 1,300 ppm of benzene (**V_(H)_** = 1.3 V, **V_(G)_** = 6 V), and stay unchanging vs. the humidity.

## 6. Conclusions

In this paper we modeled three TGS8xx sensors using an artificial neural network sensor. It accurately reproduces the behavior of the gas sensors in a dynamic environment by taking into account the nonlinearity of their responses, the dependence on temperature and relative humidity at the measurement point, in addition to the dependence on the gas nature. The proposed ANN models were tested and implemented as components of the PSPICE simulator library. We used the ANN capability to correct the nonlinear response and to compensate gor temperature and relative humidity influences on the target sensor variations which is tested by Matlab and PSPICE simulation results. We wire all the components with the selective module with the aim of increasing the selectivity. The new technique discriminates and qualitatively and quantitatively measured seven gase,s tested between 300 and 5,000 ppm, with few sensors and this method can be easily extended to other kinds of sensors.

## Figures and Tables

**Table 1.** Summary the model's optimized parameters.

**Property**	**Characteristic**	
Database	Training base	3,800
	Test base	504
Architecture	9-5-1 Feed-forward MLP	
Activation functions	Logsig-Logsig- linear	
Training rule	Retropropagation error	
Training MSE	<0.0001	
Iterations number	3,000	

**Table 2.** Summary of the corrector's optimized parameters.

**Property**	**Characteristic**	
Database	Training base	3,800
	Test base	504
Architecture	9-8-1 Feed-forward MLP	
Activation functions	Logsig-Logsig- linear	
Training rule	Retropropagation error	
Training MSE	<0.00001	
Iterations number	5,000	

**Table 3.** Voltages and their matched gases.

**Gas**	**Voltage [V]**
Air	1
Methane	2
CO	3
Isobutane	4
Ethanol	5
Benzene	6
*n*-Hexane	7
Acetone	8
Hydrogen	9
Propane	10

**Table 4.** Summarizing of the optimized parameters of selective module.

**Property**	**Characteristic**	
Database	Training base	250
	Test base	50
Architecture	5-2-2 Feed-forward MLP	
Activation functions	Logsig-Logsig- linear	
Training rule	Retropropagation error	
Training MSE	<10^−6^	
Iterations number	1,000	
